# Optimization of a Pre-MEKC Separation SPE Procedure for Steroid Molecules in Human Urine Samples

**DOI:** 10.3390/molecules181114013

**Published:** 2013-11-13

**Authors:** Ilona Olędzka, Piotr Kowalski, Szymon Dziomba, Piotr Szmudanowski, Tomasz Bączek

**Affiliations:** Department of Pharmaceutical Chemistry, Medical University of Gdansk, Hallera 107, 80-416 Gdańsk, Poland

**Keywords:** human urine samples, MEKC technique, steroid hormones

## Abstract

Many steroid hormones can be considered as potential biomarkers and their determination in body fluids can create opportunities for the rapid diagnosis of many diseases and disorders of the human body. Most existing methods for the determination of steroids are usually time- and labor-consuming and quite costly. Therefore, the aim of analytical laboratories is to develop a new, relatively low-cost and rapid implementation methodology for their determination in biological samples. Due to the fact that there is little literature data on concentrations of steroid hormones in urine samples, we have made attempts at the electrophoretic determination of these compounds. For this purpose, an extraction procedure for the optimized separation and simultaneous determination of seven steroid hormones in urine samples has been investigated. The isolation of analytes from biological samples was performed by liquid-liquid extraction (LLE) with dichloromethane and compared to solid phase extraction (SPE) with C_18_ and hydrophilic-lipophilic balance (HLB) columns. To separate all the analytes a micellar electrokinetic capillary chromatography (MECK) technique was employed. For full separation of all the analytes a running buffer (pH 9.2), composed of 10 mM sodium tetraborate decahydrate (borax), 50 mM sodium dodecyl sulfate (SDS), and 10% methanol was selected. The methodology developed in this work for the determination of steroid hormones meets all the requirements of analytical methods. The applicability of the method has been confirmed for the analysis of urine samples collected from volunteers—both men and women (students, amateur bodybuilders, using and not applying steroid doping). The data obtained during this work can be successfully used for further research on the determination of steroid hormones in urine samples.

## 1. Introduction

Steroid hormones are a group of natural compounds whose structure is based on their carbon skeleton being a derivative of 1,2-cyclopentanoperhydrophenanthrene. Despite the fact that different classes of steroid hormones have different chemical structures and functions, they have a similar mechanisms of action that involve the direct regulation of the rate of transcription of the corresponding genes [1]. A reliable determination of the levels of steroid hormones in the human body and biological fluids is an essential diagnostic tool. The choice of material for such biological studies is also critical. The most widely used biological materials for the investigation of these hormones are blood, urine and saliva samples [2,3,4,5]. The determination of steroid hormone levels in blood samples may be impaired due to the oscillatory secretion of these compounds according to the circadian rhythm and the menstrual cycle. Saliva as a biological material is characterized by a low invasiveness sampling method, but the concentrations of steroid hormones in this matrix are about 1,000–5,000 lower than in blood. On the other hand, saliva analysis is very important due to the fact that it allows an evaluation of the free fraction of the hormones that are not bound to protein [3,4]. On the other hand, metabolites of most steroid hormones produced in the body are excreted with urine, which allows the assessment of full hormonal profiles. The analysis of steroid hormones in urine samples is a very significant and important diagnostic tool because it enables the activity of several endocrine organs (hypothalamus, pituitary, the endocrine glands) to be determined so measurement of the concentration of steroid hormones is an important endocrine diagnosis of many diseases [5]. Moreover, it is the most effective tool for the detection of illegal doping in sport. As it is known, anabolic steroids, which belong to the synthetic derivatives of testosterone, are the main substances used in pharmacological doping. Modifications of the testosterone molecule increase its anabolic properties compared with its androgenic action [3,6].

The most common methods used for the quantification of steroid hormones in urine samples are immunological methods [7,8], high-performance liquid chromatography (HPLC) [6,9] or gas chromatography techniques (GC) [10]. Generally, immunological techniques are characterized by speed, however, many authors emphasize that most applications based on immunoassay are not specific for steroid determination. Very often this method does not measure steroid hormones alone, but also the interfering material with apparent metabolites or their derivatives [11]. On the other hand, despite the fact that the HPLC methods do not have any cross-reactivity between the various steroids, it is often seen as having low resolution and needing a large volume of eluents. In doping control, enzymatic methods (EIA) and radioimmunoassay (RIA) using radiolabeled antibodies are used routinely [12]. Likewise, in order to perform more precise tests, techniques based on GC/MS/MS, GC/C/IRMS (gas chromatograph with a combustion chamber-C-isotope mass spectrometer-IRMS) have been developed [13]. The GC/C/IRMS method is used to detect synthetic and endogenous androgens, especially when the ratio of testosterone and epitestosterone (T/ET) is greater than 6, but does not exceed a value of 10. However, the mentioned methods require a substantial financial investment and in addition are both time-and labor-intensive. Capillary electrophoresis (CE) may be an excellent alternative to the discussed techniques, since it is a simple and reliable analytical tool with high resolution, and relatively short separation times. Another advantage of CE is the very small volume of the sample used (on the order of nL) and the possibility of separating electrically neutral compounds along with charged compounds.

As regards how to extract the steroids from biological materials, the available literature proposes numerous options. As shown by many authors, the evaluation of steroid hormones in urine often causes some problems, due first of all to the presence of other hormonal substances and their metabolites, with similar hydrophobicity. Furthermore, they may be present in high concentrations and interfere with the signals from the analytes of interest. As can be seen from the literature, researchers studying the determination of steroid hormones have employed different extraction methods: liquid-liquid extraction (LLE) [14,15,16,17,18], solid phase extraction (SPE) [19,20,21,22,23] or solid phase microextraction (SPME) [4].

Therefore, the aim of this study was to develop electrophoretic methods for the identification and determination of steroid hormones in urine samples of volunteers and amateur weight-lifters by the micellar electrokinetic capillary chromatography (MEKC) technique. We have provided a new strategy for solving the problem of sample treatment when endo- and exogenous steroid hormones are simultaneously determined in biological samples. Moreover, the goal of the study was also to demonstrate the application of the elaborated method for routine doping control and the quantification of steroid hormones in human urine samples. Generally, the manual sample preparation steps (e.g., multiple extraction) have been eliminated and the time required for sample processing has been greatly shortened compared to other laboratory techniques.

## 2. Results and Discussion

The quantification of small amounts of endogenous and exogenous molecules in human urine is relatively difficult to accomplish and can cause complications in the sample treatment process prior to analysis. This is due, firstly, to the complexity of the biological matrix, and secondly because of the generally very low concentrations of hormones. In most cases, it is necessary to develop an effective and efficient procedure for the preparation of urine samples for high performance separation methods.

### 2.1. Comparison and Optimization of the Extraction Procedure

In this study, based also on literature data, the effectiveness of extraction procedures of steroid hormones from urine samples was assessed. The most commonly used extraction technique is the LLE one which utilizes the distribution of the analyte(s) of interest between water and an organic solvent. However, this technique has drawbacks due to its significant consumption of solvents and a comparatively low concentration coefficient. LLE requires the use of very clean organic solvents with low solubility in water. The purity of the solvent is essential because during the pre-concentration process the concentration of any impurities increases. This type of extraction during the analysis of steroids has been employed by many researchers [14,15,16,17,18]. The main conclusions of these studies were that the extracts obtained were often found to be “too dirty” for further analysis, acidic or basic wash steps had to be introduced to remove any interferences, large volumes of organic solvents were needed and analyte recoveries were frequently very low. The extraction procedures used in this study, which were based on LLE (Procedures 1 and 2), proved to not be efficient enough for the isolation of steroid hormones from urine samples. In addition, there were a lot of signals from the sample matrix which interfered with the analyte peaks. On the other hand, SPE is often used as an extraction technique for both volatile and non-volatile analytes due to the possibility it offers to concentrate them; it allows the test compounds to be stored on the sorbent for a long time, reduces the creation of emulsions as is the case with LLE and in particular it greatly reduces the use of organic solvents. As follows from literature data, SPE extractions using C_8_ or C_18_ cartridges were frequently developed for the extraction of steroid hormones from biological samples [19]. Even better results were obtained using hydrophilic-lipophilic balance (HLB) cartridges, which are commonly applied for the extraction of a variety of analytes from water.

AbuRuz *et al.* [22] used in their research this type of cartridges for the extraction of prednisolone and cortisol from plasma and urine samples, observing high recoveries of analytes ranging between 85.4% and 101.3%. Hu *et al.* [24] described an extraction procedure for corticosteroid hormones based on HLB cartridges and found satisfactory recoveries of more than 90% for cortisol and cortisone and 70.8% for 6β-hydroxycortisol. This is caused by the comparatively higher water solubility of the latter compared to the parent molecules. Cho *et al.* [23] assessed HLB cartridges containing a copolymer sorbent that has hydrophilic and lipophilic groups and provided high recoveries for 21 endogenous corticosteroids.

**Figure 1:**
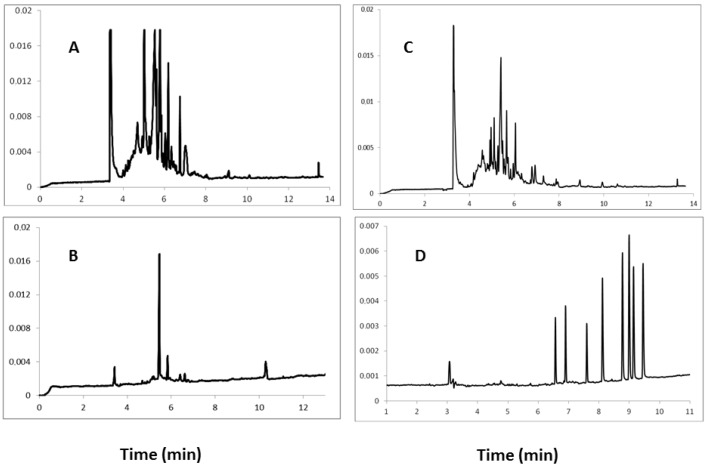
Electropherograms obtained during the electrophoretic separation of human urine samples containing eight steroid hormones after SPE using C_18_ cartridges and elution of steroids with: methanol (**A**), and dichloromethane (**B**), next SPE using HLB cartridges and elution of steroids with methanol (**C**), dichloromethane (**D**). Conditions: UV detection at 254 nm, unmodified silica capillary (57 cm × 50 μm I.D.), temp. 22 °C, running buffer composed of (10:90, v/v) methanol and mixture of 10 mM Na_2_B_4_O_7_ and 50 mM SDS.

In the present study, extraction procedures based on SPE using both C_18_ and HLB cartridges have been tested and compared. Optimal results were obtained using HLB cartridges and elution with dichloromethane. Moreover, it was further found that flushing the applied sample before its elution with a 50:50 (v/v) acetone-water mixture can significantly reduce the amount of impurities. From an electrophoretic viewpoint it has been confirmed that the extraction procedure is most effective for the isolation of analytes and removal of contaminant substances from urine samples. Electropherograms collected and obtained by SPE using both C_18_ and HLB columns as well as the eluents methanol and dichloromethane are shown in Figure 1.

### 2.2. Optimization of Electrophoretic Conditions

In order to establish optimal conditions for the simultaneous determination of all analytes the appropriate parameters of electrophoretic separation have been tested: the composition of the running buffer and the concentration of buffer components, the inner diameter and length of the capillary, the injection time, the analytical wavelength, the temperature of the system and the polarity of the electrodes.

#### 2.2.1. Separation Buffer

One of the most important parameters for the optimization procedure was the selection of a suitable running buffer for the effective separation of all components. The appropriate choice of the type of buffer, the ionic strength (concentration), and the pH value can have a major impact on the quality and efficiency of electrophoretic separations. For the determination of steroid hormones alkaline buffers containing borax [1,25], ammonium acetate [21], sodium cholate [16] and containing small amounts of methanol (up to 20%) in each buffer have been mostly used. Kartsova *et al.* [25] conducted a comparative study on the impact of the pH of the running buffer on the separation of steroid hormones by MEKC. The application of the reversed polarity of the electrodes and acidic buffer (pH = 2.5) allows for the separation of nine analytes while the use of the normal polarity and alkaline buffer (pH = 9.2) enabled the separation of only seven steroid hormones. The addition of urea to the buffer gave rise to hydrogen bonds with the SDS, and weakened hydrophobic interactions between analytes and the surfactant, resulting in increased separation efficiency. Moreover, the conditions applied by Kartsova *et al.* [25] led to increased electrolyte viscosity. Due to the very low pH of the electrophoretic buffer the authors used reversed polarity electrodes, so the analysis time was significantly reduced. Table 1 shows the composition and the effect of the running buffers employed in this study. During the optimization procedure, borate buffers, which exhibit an alkaline pH value and a relatively low ionic strength were mainly used. The optimal background electrolyte was the buffer containing 10 mM borax, 50 mM SDS, and 10% methanol. The addition of an organic modifier was necessary to achieve full separation of all the analytes and prevent overlapping peaks while also providing the optimal current intensity order of 45 μA at an applied voltage of 24 kV. The other important compound of the background electrolyte was the surfactant. Numerous surfactants have been developed for the separation of steroid hormones by MEKC, such as anionic [25,26,27], biologically active [16], cationic [28] and microemulsion pseudostationary phases [29]. The most popular anionic modifier is sodium dodecyl sulphate (SDS), which can form micelles when the concentration of the surfactant exceeds the critical micelle concentration (CMC). The formation of such micelles can increase the solubility of poorly soluble compounds (micellar solubilization) and also provides the opportunity to separate molecules without charge (such as neutral substances). All of these properties are useful for the successful determination of steroid hormones because they are neutral and hydrophobic molecules.

**Table 1:** The composition of tested buffers and their effect on the electrophoretic view.

Buffer composition	Observations
10 mM Na_2_B_4_O_7_, 50 mM SDS + 20% MeOH	Satisfactory separation was achieved with applied voltage 23 kV, generated current 45–50 μA. Relatively long analysis time; high and sharp peaks of analytes; the phenomenon of “tailing” peaks caused by too high content of methanol.
20 mM Na_2_B_4_O_7_, 50 mM SDS + 20% MeOH	Separation was deemed unsatisfactory, peaks with a broad base, interfering with each other, just as in the previous test buffer phenomenon of “tailing”, the current slightly higher than in the buffer before test. The analysis time was shortened slightly.
10 mM Na_2_B_4_O_7_, 50 mM SDS	The peaks were very narrow, but overlapping. During the analysis of eight analyte standards five overlapping peaks were obtained within a short analysis time.
10 mM Na_2_B_4_O_7_, 50 mM SDS + 15% MeOH	The peaks were very sharp, well-separated from each other, but a relatively long analysis time (16 min) was necessary.
10 mM Na_2_B_4_O_7_, 50 mM SDS + 10% MeOH	The peaks were very sharp, narrow, symmetrical, non-overlapping, and excellently separated. This buffer proved to be the most optimal separation electrolyte.
10 mM NaH_2_PO_4_, 25 mM SDS (pH 3.0)	Separation was not achieved after 30 min.

#### 2.2.2. Capillary Parameters and Conditioning

Another crucial step in the optimization of the electrophoretic separations was the selection of the optimal capillary parameters. For this purpose unmodified silica capillary tubes of different lengths (47–77 cm) and internal diameters (50–100 μm) were tested [16,25,30]. The choice of the inner diameter and the length of the capillary has a very significant impact on the effectiveness of electrophoretic separations. In this case, the optimal capillary has proven to be an unmodified silica capillary of 57cm length and of 50 μm inner width. To ensure repeatability of the analysis, after each separation process the capillary was regenerated by rinsing with appropriate solutions: 0.1 N NaOH (regenerating solution), methanol (used to clean the capillary impurity residues, derived from biological samples), deionized water (washing all the previously used compounds). In addition, the capillary was conditioned daily before the analysis by flushing with 0.1 N NaOH (the aim is to facilitate the ionization of silanol groups on the surface of the inner capillary wall) for 1 min, then methanol for 3 min, and finally deionized water for 3 min.

#### 2.2.3. Injection

Hydrodynamic injection mode was selected to introduce the sample into the capillary. In order to select the most optimal separation conditions the appropriate injection time should be established, which affects the quality of separation. Too long an injection time may cause an “overload of the capillary”, which can result in the appearance of broad, high and overlapping peaks. On the other hand, too short an injection time can result in a too small amount of the sample injected into the capillary, thereby reducing the peak height or creating the absence of signals. In our study we investigated the effect of the injection time in a range of 2 to 15 s. After applying 2 s and 5 s injection times, the obtained peaks were sharp, very slim and symmetrical. There were no interference peaks or evidence of the “tailing peaks” phenomenon. When applying the 10 s injection time the peaks overlapped, their base was wide, and the shape was less sharp than with the 5 s injection. In addition, there was the phenomenon of “tailing peaks.” With further increases of the injection time the peaks of interest were significantly broadened.

#### 2.2.4. Analytical Wavelengths

**Figure 2:**
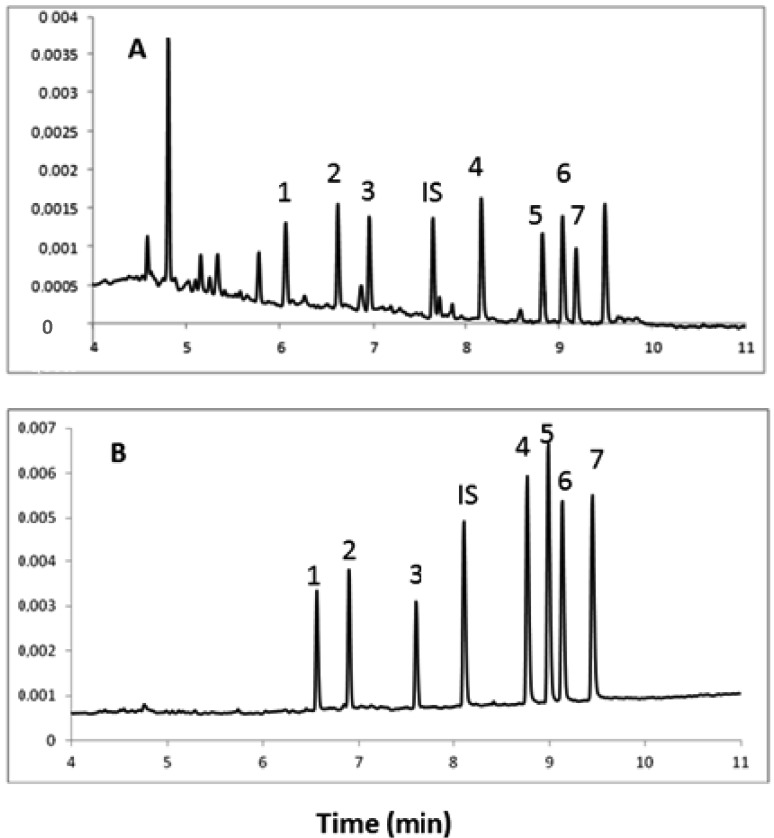
Electropherograms obtained during the electrophoretic separation of the urine sample containing 8 steroid hormones analyzed at λ = 200 nm (**A**) and 254 nm (**B**). Other conditions the same as in Figure 1. Analytes: 1—cortisone, 2—cortisol, 3—corticosterone, IS—dexamethasone, 4—testosterone, 5—17α-MT, 6—epitestosterone, 7—progesterone.

As shown in the literature, steroid compounds, including doping substances, are determined using two wavelengths, at which the analyzed compounds exhibit absorption maxima (λ = 200 nm and λ = 254 nm) [31]. In this paper, a simple UV detector at a wavelength of 254 nm was performed. Electropherograms obtained during the electrophoretic separation of urine samples containing eight steroid hormones analyzed at λ = 200 nm (A) and 254 nm (B) are presented in Figure 2. During the CE separation of urine samples at 200 nm, it was found that the impurity residues of biological samples showed relatively strong interactions with the analyte peaks. However, the analysis carried out at the analytical wavelength λ = 254 nm proved to be the most optimal, because at this wavelength no interference of interfering substances with the analyte peaks were observed.

#### 2.2.5. Temperature of Analysis

Another important parameter in the electrophoretic analysis is the temperature. The temperature has an influence on the density and the mobility of ions, as well as on the volume of the injected sample. The effect of separation was compared at different temperatures, namely 20–26 °C. The most optimal separation conditions prevailed when a temperature of 22 °C was employed, therefore all separations were carried out at 22 °C.

#### 2.2.6. Voltage

A very important parameter in the electrophoretic analysis is the applied voltage. It is also a parameter that could be modified very easily and quickly and the effect of changes observed. Changing the voltage up to 1 kV not causeed significant differences in the electrophoretic view. During the optimization of the CE method a voltage range of 18–25 kV was estimated. The most optimum separation was achieved when a voltage of 24 kV was applied. A lower voltage caused the separation time to be longer and the phenomenon of “tailing peaks” appeared.

### 2.3. Validation of the Method

Validation is the process which can accurately establish the inherent quality of an analytical method by the fulfillment of minimum acceptance criteria and thus verify its applicability for a certain purpose. The developed method was validated to determine the seven steroid hormones and I.S. in human urine samples. The procedure of validation was based on the ICH guidelines for analytical method validation for human studies [32].

#### 2.3.1. Specificity

**Figure 3:**
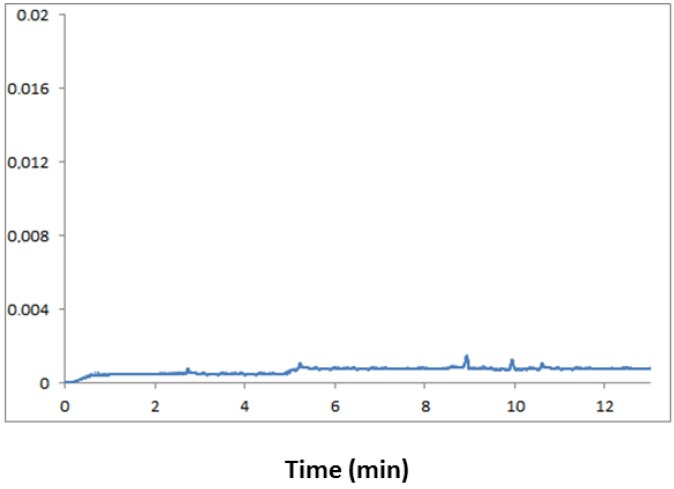
Representative electropherogram of a blank urine sample obtained after the SPE clean-up procedure. All separation conditions the same as in Figure 1.

One approach to establishing method specificity is to demonstrate a lack of response in a blank matrix so that there are no signals interfering with the signal of the analytes and I.S. The specificity of the elaborated method was estimated by comparing the electropherograms of the blank urine samples with the urine samples fortified with the analyzed hormones and the internal standard (n = 6). The electropherogram of the blank urine samples, collected from volunteers, is presented in Figure 3. During the separation process no interferences were observed at the migration time of the analytes of interest from the endogenous urine components. Therefore, it was concluded that the specificity of the method was confirmed.

#### 2.3.2. Linearity

Linearity is an important calibration parameter because it examines the relationship between the analyte concentration in the sample and the corresponding response of the measurement system. The development of methods for separating seven steroid hormones requires the preparation of a calibration curve for each analyte. To achieve this goal blank urine samples (5 mL) were spiked with increasing concentrations (5, 10, 25, 50, 100, 250, 500 and 1,000 ng mL^−1^) of cortisone, cortisol, corticosterone, testosterone, 17α-methyltestosterone, and epitestosterone and an internal standard (dexamethasone, 500 ng mL^−1^). For progesterone the spiked concentrations were 25, 50, 75, 100, 150, 250, 500, and 1,000 ng mL^−1^ and dexamethasone in an amount of 500 ng mL^−1^.

Samples prepared in this manner were then subjected to SPE according to procedures described in Section 3.3. After being dissolved in 25 µL of methanol, and 225 µL of 2 mM borax solution the samples were analyzed by capillary electrophoresis. The corresponding calibration parameters of the seven analytes are summarized in Table 2. For each analyte of interest, the calibration curve designated the correlation coefficient (*r*^2^) from 0.9995 for progesterone to 0.9999 for cortisol and cortisone, which indicates excellent linearity of the elaborated method in the considered concentration range of 5–1,000 ng mL^−1^.

**Table 2:** Linearity, detection and quantitation limits determined for the proposed MEKC method for steroid hormones.

Compound	Intercept (±SD)	Slope (±SD)	r^2^	LOD [ng mL^−1^]	LOQ [ng mL^−1^]
Cortisone	0.0025 (7.1 × 10^−6^)	0.0417 (0.0029)	0.9999	1.5	5
Cortisol	0.0035 (2.2 × 10^−5^)	0.0163 (0.0091)	0.9999	1.5	5
Corticosterone	0.0024 (2.0 × 10^−5^)	0.0961 (0.008)	0.9996	1.5	5
Testosterone	0.0029 (1.9 × 10^−5^)	0.079 (0.0078)	0.9997	1.5	5
17α-Methyltestosterone	0.0029 (1.6 × 10^−5^)	0.0617 (0.0066)	0.9998	1.5	5
Epitestosterone	0.004 (3.1 × 10^−5^)	−0.0008 (0.0125)	0.9996	1.5	5
Progesterone	0.0021 (2.4 × 10^−5^)	0.0056(0.0113)	0.9995	7	25

#### 2.3.3. Limits of Detection and Limit of Quantitation

The limits of detection (LOD) were established by six-fold replicate separation and were specified to be 1.5 ng mL^−1^ for androgen and corticosteroids, and 7.0 ng mL^−1^ for progesterone. The limit of quantitation (LOQ) value for androgens and corticosteroids, is 5 ng mL^−1^, and for progesterone 25 ng mL^−1^. The achieved LOD and LOQ values in the presented method were lower when compared with the earlier published MEKC assays with UV detection [16,19].

#### 2.3.4. Precision

Intraday (n = 6) and interday (n = 9) precision values of the procedure for all the analyzed hormones are presented in Table 3.

**Table 3:** Intra-assay precision, inter-day precision, and recovery at three concentration levels for steroid hormones.

Steroid Nominal concentration [ng mL^−1^]	Intra-day precision (n = 6)		Inter-day precision (n = 6)		Recovery (%)
	Concentration found [ng mL^−1^] (±SD)	RSD (%)	Concentration found [ng mL^−1^] (±SD)	RSD (%)	
**Cortisone**					
10	10.1 (±0.7)	7.0	10.7 (±1.1)	8.5	100.5
100	103.0(±3.4)	3.3	105.1 (±4.1)	3.9	103.0
500	493.6 (±6.0)	1.2	509.0 (±4.9)	1.0	98.7
**Cortisol**					
10	11.4 (±0.6)	5.1	10.9 (±0.9)	8.4	114.0
100	101.4 (±2.9)	2.9	106.3 (±4.5)	4.2	101.4
500	497.5 (±10.5)	2.1	505.1 (±5.9)	1.2	99.5
**Corticosterone**					
10	11.0 (±0.9)	8.3	11.3 (±1.0)	8.8	110.3
100	108.4 (±8.4)	7.8	91.1 (±7.1)	7.9	108.4
500	502.0 (±1.3)	0.3	507.1 (±4.5)	0.9	100.4
**Testosterone**					
10	9.9 (±0.9)	9.1	10.4 (±1.0)	9.5	99.1
100	97.9 (±4.7)	4.8	93.7 (±6.2)	6.6	97.9
500	498.9 (±6.3)	1.3	504.4 (±7.3)	1.4	99.8
**17α-Methylotestosterone**					
10	10.9 (±1.0)	9.2	11.1 (±1.1)	9.7	108.6
100	102.2 (±5.7)	5.6	106.1 (±7.1)	6.7	102.2
500	497.7 (±10.7)	2.2	507.3 (±18.5)	3.7	99.5
**Epitestosterone**					
10	10.7 (±1.0)	9.2	10.8 (±1.0)	9.2	106.8
100	108.8 (±5.3)	4.9	109.3 (±6.1)	5.6	108.8
500	502.9 (±17.6)	3.5	509.7 (±19.5)	3.8	100.6
**Progesterone**					
25	28.6 (±2.5)	8.7	29.8 (±2.9)	9.7	114.2
100	101.8 (±5.4)	5.3	106.4 (±6.2)	5.8	101.8
500	486.0 (±18.4)	3.8	510.6 (±19.5)	3.8	97.2

The intraday precision expressed as the RSD ranged between 0.3% for corticosterone to 9.2% for 17α-methyltestosterone and epitestosterone. In turn, the interday precision data went from 0.9% (corticosterone) to 9.7% (17α-methyltestosterone and progesterone). None of the evaluated RSD values exceeded 10%. According to the Food and Drug Administration (FDA) guidelines the acceptance criteria for precision depends mostly on the type of analysis. The precision value for biological samples stands at ca. 15% at the concentration limits and at 10% at other concentration levels. The data obtained in this experiment indicated that each analyte met the generally accepted criteria for validation by the bioanalytical method at all calibrations and the quality control (QC) concentration levels.

#### 2.3.5. Recovery

Although many authors consider that the determination of the recovery is not as important as the designation of LOD, precision and accuracy [32], in this study, the recoveries for all analytes were estimated. Recoveries achieved with the selected SPE procedure are presented in Table 3. The obtained recovery results ranged from 98.7% to 103.0% for cortisone, 99.5%–114.2% for cortisol, 100.4%–110.3% for corticosterone, 97.9%–99.8% for testosterone, 99.5%–108.6% for 17α-methyltestosterone, 100.6%–108.8% for epitestosterone and 97.2%–114.2% for progesterone.

#### 2.3.6. Stability

According to the guidelines given by Peters *et al.* [32] full validation of the method must include the stability of the analyte in the sample matrix and for this tests should be established. A stability test of the elaborated method was carried out successfully. It was based on the re-measurement of the concentration of analytes in the same samples “*ex tempore*” and after 1 and 3 months after storage at ‒20 °C. After comparing the results, one month after storage recovery values were in the range 97.4%–100.2%, the average value was 98.2%, and after 3 months, the determined values were within 93.1%–100.9% and the average value was 95.3%.

### 2.4. Application of Human Urine Samples

As an application of the elaborated method, urine samples from volunteers: amateur weight-lifters using hormonal doping (13 subjects), amateur weight-lifters not using hormonal doping (three) and from healthy volunteers (five) were collected and investigated, taking into account the level of some steroids.

There are many reasons for an abnormal secretion of androgens. In men, it is usually hypogonadotropic hypogonadism (secondary) and hypergonadotropic hypogonadism (primary). In children, it is delayed or precocious puberty. In women, the most common cause is polycystic ovary syndrome. These are serious disorders, which may significantly reduce the quality of life. Testosterone, because of its anabolic effects, is one of the basic and most used anabolic steroids in doping. In a short time, it causes a significant increase of muscle mass and strength. Testosterone and its derivatives are used mostly by athletes who want to quickly improve their performance or sports enthusiasts who want to quickly build muscle mass [3,33]. For the purposes of doping, derivatives of testosterone and dihydrotestosterone are used to increase muscle mass, increase muscle strength and improve overall performance. Often several hormonal substances are used at the same time to improve the desired effect, but the serious side effects also increase too. Epitestosterone was used as a doping agent to mask the excessive use of synthetic testosterone (which should not exceed the limit of the T/ET ratio). However, this treatment is not effective, since the anti-doping analysis in the study included the simultaneous quantification of both hormones. The doping control test is the most appropriate indication of the ratio of testosterone glucuronide to epitestostrone glucuronide in urine. The limit of this ratio is 4. Methyltestosterone was also used in doping, as an anabolic agent and to enhancd the ability to fight in sports and even aggression, making it especially popular among people practicing martial arts, due to the increased aggression and the anabolic effects [13]. The concentration of cortisol is usually determined based on urine or serum levels. The advantage of the determination of this hormone in urine is the non-invasive sampling. The most reliable test is the analysis of hydrocortisone in daily urine collection, in which the average concentration of free cortisol is estimated. Because cortisol levels rapidly change under the influence of stress factors, this hormone has become an important biomarker of stress exposure. Long-term secretion of large amounts of cortisol by the human body in response to chronic stress may lead to Cushing’s syndrome. In addition, the assessment of the cortisol levels can help diagnose many other diseases. Pathological conditions in which there is a disorder in cortisol levels include primary and secondary adrenal insufficiency (Addison’s disease), Cushing’s syndrome (hypercortisolism), secondary adrenal overactive, disorders of the hypothalamic-pituitary-adrenal axis, hirsutism, overtraining in athletes (ratio of free testosterone to cortisol). Hypercortisolism is frequently associated not only with an excess of glucocorticoid effects, but also with an increase in mineralocorticoid activity. The cortisol/cortisone ratio is particularly elevated in patients with ectopic adrenocorticotropic hormone (ACTH) syndrome [17].

Table 4, Table 5 and Table 6 show the results of concentrations of steroid hormones in urine obtained in this study. The level of testosterone (T) excreted in the urine under physiological conditions is generally in the range of 20–150 ng mL^−1^, and in people who practice various sports, especially power sports, the level may exceed 150 ng mL^−1^. Same number In our study in patients taking exogenous steroid hormones abnormally high levels of testosterone (a mean of 2.300 ± 1.141 ng mL^−1^), with a mean epitestosterone (ET) concentration of 225.1 ± 61.1 ng mL^−1^ were observed and the T/ET ratios were in any case exceeded (mean 10.0 ± 3.6) (Table 4). In the case of persons practicing strength sports, but not using hormonal doping the level of urinary excretion of testosterone was in the physiological range with a mean of 81.7 ± 5.3 ng mL^−1^; the mean epitestosterone was 41.3 ± 8.7 ng mL^−1^ and the mean T/ET ratio was 2.05 ± 0.6 (averaged data from Table 5).

**Table 4:** The values of concentrations of steroid hormones (in ng mL^−1^) identified in urine samples of amateur weight-lifters using hormonal doping.

Subject	Cortisone	Cortisol	Corticosterone	Testosterone	17α-MT	Epitestosterone	Progesterone	T/ET
1	181.8	170.7	57.3	1668.6	220.4	187.8	n.a.	8.88
2	222.4	228.3	90.2	1432.7	211.1	182.0	34.2	7.87
3	113.8	211.5	67.5	4940.9	211.1	355.2	91.1	13.9
4	167.9	269.7	64.1	2691.7	122.7	253.8	n.a.	10.6
5	153.0	260.1	47.2	1241.7	184.2	138.5	n.a.	8.96
6	145.6	234.1	65.0	2012.9	144.6	215.5	69.8	9.34
7	181.6	349.9	26.5	2371.3	108.4	223.3	144.5	10.61
8	151.3	548.3	75.5	3377.2	209.8	259.2	n.a.	13.03
9	231.9	553.1	100.4	3652.7	109.3	331.3	58.1	11.02
10	197.9	476.7	121.3	2879.1	210.4	281.2	n.a.	10.23
11	210.8	320.1	374.2	1401.5	181.9	209.9	160.3	6.67
12	209.3	318.0	129.2	1006.7	197.7	199.3	n.a.	5.05
13	267.1	501.8	100.5	2624.2	164.9	184.4	103.8	14.23

n.a. (not analyzed).

In turn, many factors may affect the level of urinary excretion of epitestosterone such as alcohol consumption, weight training, race (white, yellow, red), smoking, *etc.* [16,20], nevertheless, its concentration in urine is always within physiological limits. The T/ET ratio in healthy adults is generally close to the value of 1.2:1, and in people who train it can reach a value of up to a ratio of 4:1, but this limit should never be exceeded. Our results indicated that, in patients using hormonal doping the T/ET parameter was exceeded considerably, and was in the range of 3.2–16.6 ng mL^−1^. The concentrations of steroid hormones estimated in the urine of healthy volunteers (students) and amateur gym-goers, not using hormonal doping, were within physiological limits (Table 5 and Table 6).

**Table 5:** The values of concentrations of steroid hormones (in ng mL^−1^) identified in urine samples of amateur weight-lifters, not using hormonal doping.

Subject	Cortisone	Cortisol	Corticosterone	Testosterone	17α-MT	Epitestosterone	Progesterone	T/ET
1	128.4	185.1	79.8	87.6	14.2	31.5	n.a.	2.77
2	134.9	181.9	94.2	77.6	19.8	48.0	n.a.	1.61
3	129.7	167.6	89.1	79.9	n.a.	44.4	n.a.	1.78

n.a. (not analyzed).

**Table 6:** Steroid hormone concentrations (in ng mL^−1^) designated in urine samples from healthy volunteers (students).

Subject (sex)	Cortisone	Cortisol	Corticosterone	Testosterone	17α-MT	Epitestosterone	Progesterone	T/ET
1 (M)	197.8	349.6	57.3	18.5	5.5	38.6	n.a.	0.47
2 (M)	199.5	288.3	90.2	21.3	n.a.	32.1	n.a.	0.66
3 (M)	179.8	296.8	67.5	19.6	n.a.	49.3	n.a	0.4
4 (F)	186.4	327.0	64.1	9.4	n.a.	21.2	29.2	0.44
5 (F)	213.4	279.5	47.2	11.9	n.a.	18.7	25.6	0.63

M = male; F = female; n.a. (not analyzed).

In the healthy volunteers group an increased level of cortisol (a biomarker of exposure to stress) was observed (ranging 279.5–349.6 ng mL^−1^). Furthermore, in this group only in one case was the presence of 17α–methyltestosterone (which is classified as an exogenous steroid) observed. The presence of this hormone can be explained by exposure to various factors such as methylating agents (formaldehyde) or the possible use of certain drugs, which the volunteer did not mention. In the group of sports enthusiasts using illegal performance enhancers, in which the average level of 17α-MT was 165.0 ± 45.7 ng mL^−1^, the presence of methyltestosterone can be explained by consumption or by naturally occurring testosterone metabolism [13]. In the case of people practicing strength sports who did not use doping (a mean of 11.3 ± 10.2 ng mL^−1^), the presence of this exogenous steroid may be caused by oxidative stress resulting from increased gas exchange, oxygen debt, and a lot of physical effort. All these reasons may lead to *in vivo* conversion of testosterone into 17α-methyltestosterone.

**Figure 4:**
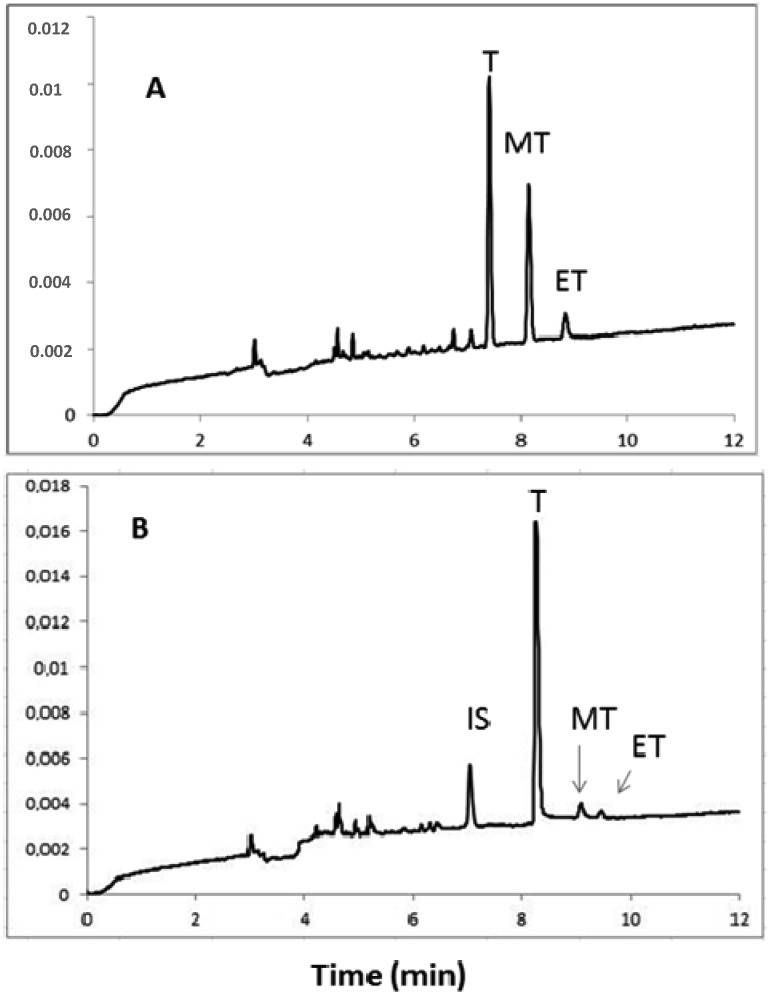
Electropherograms obtained during the electrophoretic separation of urine samples from amateurs weight-lifters, using hormonal doping. Conditions as in Figure 1. Analytes: T—testosterone, MT—17α-methyltestosterone, ET—epitestosterone, I.S.—dexamethasone.

High androgen levels and a low concentration of progesterone in men and in women is normal. A physiological phenomenon is the low level of male sex hormones such as testosterone and epitestosterone and accordingly a higher level of progesterone. Electropherograms obtained during the electrophoretic separation of urine samples from amateurs weight-lifters, using hormonal doping are presented in Figure 4.

## 3. Experimental

### 3.1. Material

Cortisol (11β,17α,21-trihydroxypregn-4-ene-3,20-dione), cortisone (17-hydroxy-11-dehydro-corticosterone), corticosterone (11β,21-dihydroxypregn-4-ene-3,20-dione), testosterone (17β-hydroxy-androst-4-en-3-one), epitestosterone (17-α-hydroxyandrost-4-en-3-one), methyltestosterone (17β-hydroxy-17α-androst-4-ene-3-one) and dexamethasone (9α-fluoro-11β,17α,21-trihydroxy-16α-methyl-pregn-1,4-dien-3,20-dione) used as the internal standard were obtained from Sigma (St. Louis, MO, USA) and were of a minimum purity of 99%. Other reagents such as dichloromethane, acetone, methanol and sodium dodecyl sulphate (SDS) were obtained from Merck (Darmstadt, Germany). β-Glucuronates was purchased from Sigma. Highly pure water was obtained from Milli-Q equipment (Millipore, Bedford, MA, USA). For the extraction of hormones from the urine samples, the following types of SPE cartridges were used: LiChrolut RP-18 (500 mg/6 mL) from Merck and hydrophilic-lipophilic balance (HLB) cartridges (200 mg, 6 mL), which were purchased from Supelco (Park Bellefonte, PA, USA).

### 3.2. Preparation of the Standard Solution of Steroids

Each of the analyzed steroid hormones was precisely weighed (at a concentration of 1 mg mL^−1^) and dissolved in methanol. Solutions prepared in this way were stored in a refrigerator at 4 °C to avoid decomposition and were diluted to an appropriate concentration (100, 10, 1 µg mL^−1^ and 100 ng mL^−1^) immediately before use

### 3.3. Isolation Procedure of Steroid Hormones from Biological Samples

To select the most effective technique for extraction of steroids from urine samples, to concentrate them, and to increase the sensitivity of the electrophoretic assays, two types of extraction systems (LLE and SPE) were tested and compared. The liquid-liquid extraction was carried out in two versions. The first procedure was the extraction of urine samples using dichloromethane with the step of acidifying the urine sample using 36% HCl. In detail the procedure was as follows: from each of the collected urine samples a volume (5 mL) was taken and next the samples were centrifuged for 7 min at 10,000 rpm. Subsequently, standard solutions of analyzed steroids were added and the samples were acidified with 36% HCl (100 µL) and mixed for 30 s. Double extraction with dichloromethane (2 × 2 mL) was carried out. The organic layer was separated and evaporated on a water bath at 45 °C in a stream of air. The residue was dissolved in a mixture composed with methanol (25 µL) and a 2 mM solution of borax (225 µL). Then the samples were thoroughly mixed for 30 s, transferred to clean Eppendorf tubes and stored at −20 °C until analysis. Procedure 2 proceed as Procedure 1 however, without the step of acidifying the urine sample using 36% HCl.

Further procedures were based on SPE extraction and varied in the type of cartridges (C_18_ or HLB) and solvent used for the elution of the analytes (methanol or dichloromethane). The procedure was as follows: from each of the collected urine samples a volume (5 mL) was taken and the samples were centrifuged for 7 min at 10,000 rpm. Like Procedure 1, standard solutions of each of the analyzed hormones were added and the samples were shaken for 30 s on a shaker point and then for 10 min using a mechanical shaker. The samples were centrifuged again for 7 min at 10,000 rpm. After the urine samples were centrifuged, the solution was rapidly passed through the SPE cartridges (Merck, LiChrolut RP-18, 500 mg or HLB), preconditioned with methanol (5 mL) and washed twice with deionized water (5 mL), using a vacuum. Before elution samples were flushed with a 50:50 (v/v) acetone-water mixture. The elution of analyzed hormones was realized using methanol or dichloromethane (2 mL). Next the organic phase was evaporated to dryness at 45 °C in a water bath and the extracted analytes were dissolved in methanol (25 µL) and a 2 mM solution of borax (225 µL), and subsequently centrifuged for 7 min at 4 500 rpm and injected into the CE system. In detail:

Procedure 3. SPE using C_18_ cartridges and the elution of steroids with 2 mL of methanol.
Procedure 4. SPE using C_18_ cartridges and the elution of steroids with 2 mL of dichloromethane.
Procedure 5. SPE using HLB cartridges and an elution of steroids with 2 mL of methanol.
Procedure 6. SPE using HLB cartridges and an elution of steroids with 2 mL of dichloromethane.

In addition, each of these procedures was preceded by hydrolysis step for 3 hours at 37 °C using β-glucuronidase (100 µL) added to 5.0 mL of urine. After the hydrolysis, the urine samples were brought back again to the ambient temperature and subjected to a further extraction process.

### 3.4. Analytical Equipment

The electrophoretic separation was performed by the CE system Beckman P/ACE 5,000 (Beckman Instruments, Fullerton, CA, USA), with a UV detection system. Likewise, the software Gold System (Beckman Instruments) allowed peak integration, data acquisition and data analysis. Samples, prepared according to Procedure 6, were introduced into the capillary using a hydrodynamic type of injection for 15 s under a pressure of 0.5 psi. The system was operated at the normal polarity (injection at the positive end of the capillary) and a constant voltage of 24 kV (current of approximately 45 μA) was applied. The dimensions of the uncoated fused-silica capillaries (Beckman Coulter) were 50 μm (the inner diameter) and 57 cm (50 cm effective length) of the total length. In order to provide constant temperature conditions during the separation process the capillary was thermostated in a circulating cooling liquid.

### 3.5. Validation Study

In order to carry out the validation process calibration curves were constructed over a range of 5–1,000 ng mL^−1^ for androgens and corticosteroids, and 25–1,000 ng mL^−1^ for progesterone based on the use of charcoal-stripped blank urine samples. For this purpose, to the urine samples (5 mL), increasing concentrations of cortisone, cortisol, corticosterone, testosterone, 17α-methyltestosterone, and epitestosterone and an internal standard (dexamethasone in an amount of 500 ng mL^−1^) were added so that the final concentrations of the analytes were: 5, 10, 25, 50, 100, 250, 500 and 1,000 ng mL^−1^. For progesterone the spiked concentrations were as follows: 25, 50, 75, 100, 150, 250, 500, and 1,000 ng mL^−1^ and dexamethasone in an amount of 500 ng mL^−1^. Urine samples prepared in this way were subjected to SPE extraction according to Procedure 6 as described in the Section 3.3. On the basis of the obtained results the curves were created by plotting the peak area ratios of the steroid to the I.S. (dexamethasone) *vs.* steroid concentrations, using the least-squares linear regression model. To assess linearity, six independent calibration curves were made over different days in the described manner. The specificity was confirmed by analyzing the blank urine and the urine spiked with analytes. The limit of detection (LOD) was estimated based on the signal to noise ratio. The limit of quantitation (LOQ) was defined as the lowest amount of an analyte in a sample which can be quantitatively estimated with suitable precision and accuracy. The intraday precision (repeatability), expressing precision under the same operating conditions over a short interval of time, was determined in six replicated analyses of the calibration control samples, at the three concentrations used to prepare the calibration plots. The interday precision was assessed on different days of the experiment over 3 months by analyzing the QC samples at concentration ranges of 10, 100 and 500 ng mL^−1^ for androgens and corticosteroids, and 25, 100 and 500 ng mL^−1^ for progesterone. The precision was expressed as the relative standard deviation (RSD). The recoveries were calculated as the percentage of each steroid response (peak areas) after the sample preparation procedure mentioned above and compared to that of non-extracted samples containing the analyte at a corresponding concentration of six independently made replications at three concentration levels, *i.e.*, 10, 100, and 500 ng mL^−1^ for androgens and corticosteroids and at 25, 100, and 500 ng mL^−1^ for progesterone. The validation procedure also comprises an evaluation of the stability of the analytes in urine after storage at −20 °C at intervals over 1 and 3 months.

### 3.6. Application of the MEKC Method for Steroid Determination in Human Urine Samples

The elaborated MEKC method was developed to estimate the levels of seven steroid hormones in human urine samples from amateur weight-lifters using hormonal doping (13 persons), amateur weight-lifters not using hormonal doping (three persons) and healthy volunteers (five persons). The research protocol was approved by the Ethical Committee from the Medical University of Gdansk. The characteristics of the persons participating in this study were as follows: amateur weight-lifters both using and not using hormonal doping ranged in ages between 23–31 years (mean ± SD; 25.8 ± 4.6), in weights from 68 to 97 kg (77.1 ± 18.3) and in height from 168–192 cm (181 ± 15.2), whereas the subjects in the student control group were aged 21–25 years (22.8 ± 1.6), weighed from 59 to 82 kg (70.1 ± 11.3) and had heights of between 165 and 187 cm (178 ± 10.2). All persons participating in this study were healthy and did not report any illnesses. The urine samples obtained from both the adult volunteers and the sports enthusiasts were frozen immediately after sampling at −20 °C until the analysis. The levels of steroid hormones were standardized by correction for creatinine excretion expressed as a steroid-to-creatinine ratio. The creatinine level in urine samples was studied using the PZ CORMAY analytical test (Lublin, Poland).

## 4. Conclusions

The methodologies used to determine steroid hormones such as corticosteroids, androgens, progestagens and their metabolites are of great importance in the field of biomedical analysis. As mentioned, the analysis of steroid hormones allows the diagnosis of many disorders ranging from stress (cortisol), through Cushing’s syndrome, Addison’s disease, hirsutism, congenital steroid enzyme deficiency, as well as enabling steroid doping tests. The results presented in this study suggest the applicability of MEKC as a separation tool allowing the simultaneous and sensitive determination of seven steroid hormones in complex matrices such as urine samples. As shown by the published literature and our earlier publications, the dedicated separation of steroid hormones by MEKC-UV [11,21,25,26,27,30], currently presented in the work allowed the achievement of a shorter time of analysis and a limit of detection sufficient for the determination of seven steroids in urine samples. Additionally, the presented SPE extraction technique based on HLB cartridges and elution with dichloromethane allows for a significant reduction of the time needed for the preparation of samples before MEKC analysis and for a reduction of impurities. The elaborated method could be proposed for clinical application because it is fast, specific, relatively cheap and accurate enough and it may be successfully applied also in the routine investigation of human urine samples. However, a specific strategy for the preparation of all biological samples has not yet been fully developed, but recently, some miniaturized devices for on-line sample extraction, and analyte preconcentration have been introduced.
